# Sinus of Valsalva Aneurysm: A Potential Case of Filamin A Mutation

**DOI:** 10.7759/cureus.45858

**Published:** 2023-09-24

**Authors:** Soe Win, Eveline Tucker, Katie Wallace, Hannah Gower, Karikalan Kandasamy

**Affiliations:** 1 Cardiology, Royal Cornwall Hospitals, Truro, GBR; 2 Emergency Medicine, Royal Cornwall Hospitals, Truro, GBR

**Keywords:** filamin a, x-linked, subependymal nodules, bentall procedure, sinus of valsalva aneurysm

## Abstract

Filamin A is a protein essential for cytoskeleton production, encoded by the X-lined dominantly inherited FLNA gene. A deficiency in filamin A can lead to cardiac valvular dysplasia and periventricular nodular heterotopia in the brain. Notably, periventricular heterotopia Type 1 has associations with cardiovascular abnormalities. We report the case of a 40-year-old woman who visited the emergency department due to shortness of breath, intermittent desaturation, and vertigo. Initial diagnostic procedures unexpectedly identified a sinus of Valsalva aneurysm on a computed tomography scan of the thorax and MRI brain revealed subependymal nodules in the lateral ventricles, suggesting an FLNA mutation. Multimodal cardiac imaging, including transesophageal echocardiogram, confirmed the aortic root aneurysm diagnosis. Consequently, the patient underwent prophylactic aortic resection and valve replacement surgery. This case underscores the importance of multidisciplinary teamwork in diagnosing and devising a comprehensive treatment plan. Cardiovascular screening for patients with known filamin A function loss might be advantageous. Similarly, genetic testing for family members could help anticipate the disease's progression and suggest prophylactic interventions like aortic root resection.

## Introduction

The aorta typically possesses three small pouches located above the aortic valve, known as the sinuses of Valsalva. A sinus of Valsalva aneurysm (SOVA), which is an enlargement of the aortic root area between the aortic valve annulus and the sinotubular ridge, is a rare cardiac malformation, occurring in 0.09% of the general population [1,2].

The FLNA gene encodes instructions for producing the filamin A protein. This protein is crucial for forming the cytoskeleton and facilitating various cellular processes, including protein movement and breakdown. Loss of function in FLNA can lead to diverse conditions, such as X-linked cardiac valvular dysplasia, periventricular nodular heterotopia in the brain, and a wide spectrum of connective tissue, lung, skeletal, and gastrointestinal disorders. Notably, periventricular heterotopia type 1 is associated with cardiovascular anomalies like patent ductus arteriosus and thoracic aortic dilatation, specifically involving the sinuses of Valsalva or the tubular aorta. This condition is X-linked dominant, predominantly affecting women with heterozygous loss-of-function variants [3].

Given the combined neurologic and cardiac pathologies observed, the following case potentially represents a variant of FLNA function loss. This case underscores the importance of comprehensive imaging modalities and the need for a multidisciplinary approach in the modern medical evaluation of patients, particularly when they present with nonspecific symptoms such as vertigo and shortness of breath.

## Case presentation

A 40-year-old woman, recently diagnosed with coronavirus disease 2019 (COVID-19), visited the emergency department due to vertigo and progressive shortness of breath upon exertion. She had a medical history of temporal lobe epilepsy and anemia. Before this visit, she had experienced exertional shortness of breath for several years, and her husband reported occasional blue discoloration of her lips. Upon physical examination, she displayed finger clubbing, slight cyanosis, elevated jugular venous pressure, and oxygen desaturation upon walking. She did not show Marfan-like features and had neither personal nor family history of connective tissue diseases.

Her vertigo and exertional desaturation were initially attributed to COVID-19. Nonetheless, a computed tomography(CT) scan of the head (CTH) and a magnetic resonance imaging (MRI) of the head (MRIH) were conducted to rule out cerebral causes of vertigo, such as a posterior stroke. At the same time, a CT pulmonary angiogram (CTPA) was performed to rule out pulmonary embolism.

**Figure 1:**
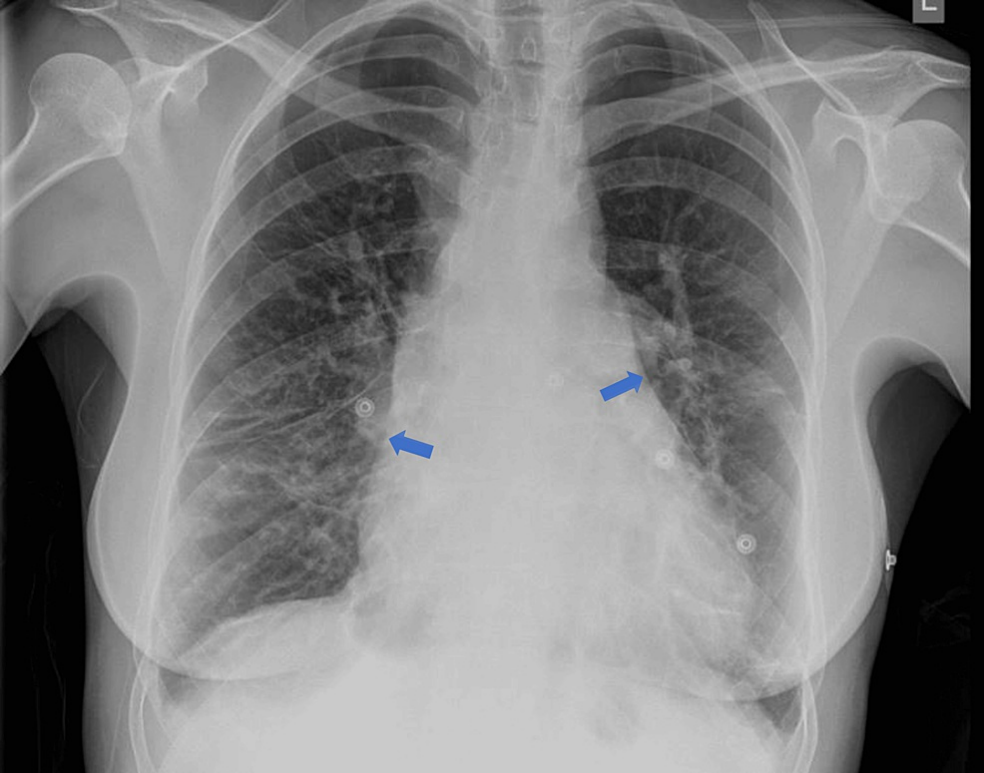
Chest X-ray demonstrating cardiomegaly and increased vasculature markings bilaterally in middle and lower zones(blue arrows)

Initial findings indicated normal liver and renal function test results and inflammatory markers were within reference ranges. However, her hemoglobin and platelet counts were low at 10 g/dL and 110 x 109/L, respectively. A chest X-ray revealed cardiomegaly and nonspecific increased lung markings in the mid and lower zones (Figure 1).

**Figure 2:**
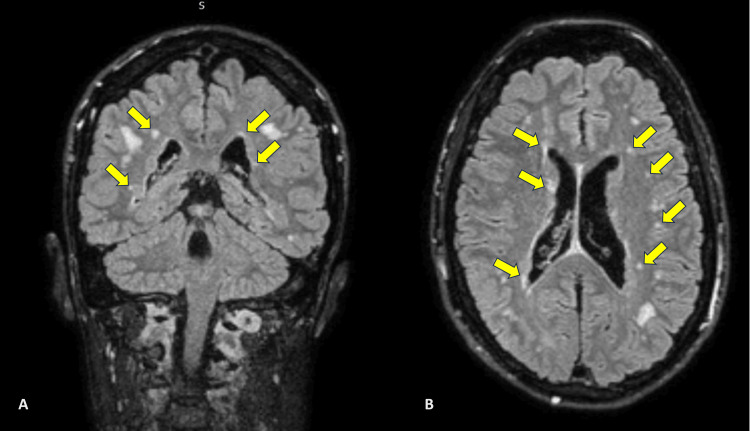
MRIH showing multiple subependymal nodules in both lateral ventricles (A, B; yellow arrows) MRIH, magnetic resonance imaging of the head

The CTH did not show acute infarction but showed slight hypoattenuation within the bilateral parieto-occipital subcortical white matter, suggesting mild to moderate vascular disease. To exclude posterior stroke, we performed an MRIH revealing multiple subependymal nodules consistent with grey matter heterotopia in both lateral ventricles without hydrocephalus. Furthermore, multifocal foci and high signal patches appeared in both cerebral hemispheres' deep cortical white matter (Figure 2).

**Figure 3:**
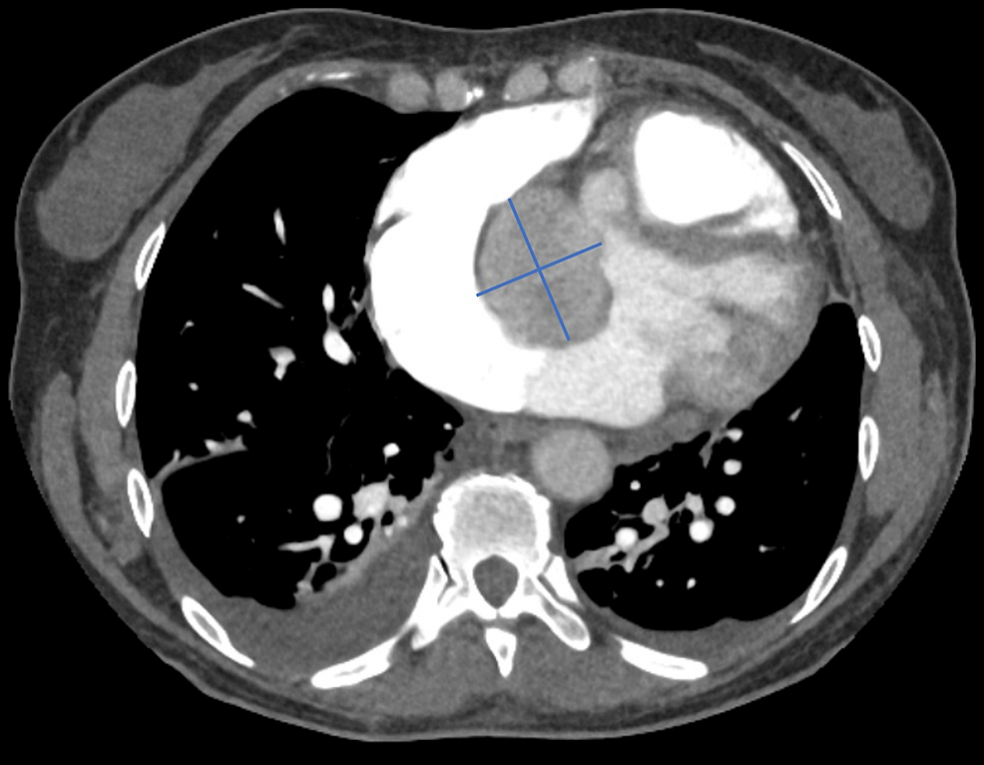
CTPA illustrating a well-defined, non-enhancing 48 mm possible cardiac mass (blue lines) CTPA, computed tomography pulmonary angiogram

CTPA ruled out pulmonary embolism but incidentally identified a 48-mm non-enhancing cardiac mass near the aortic annulus, extending into both atria. The radiologist suggested the cardiac mass might be an angiosarcoma, rhabdomyosarcoma, or myxoma (Figure 3).

**Figure 4:**
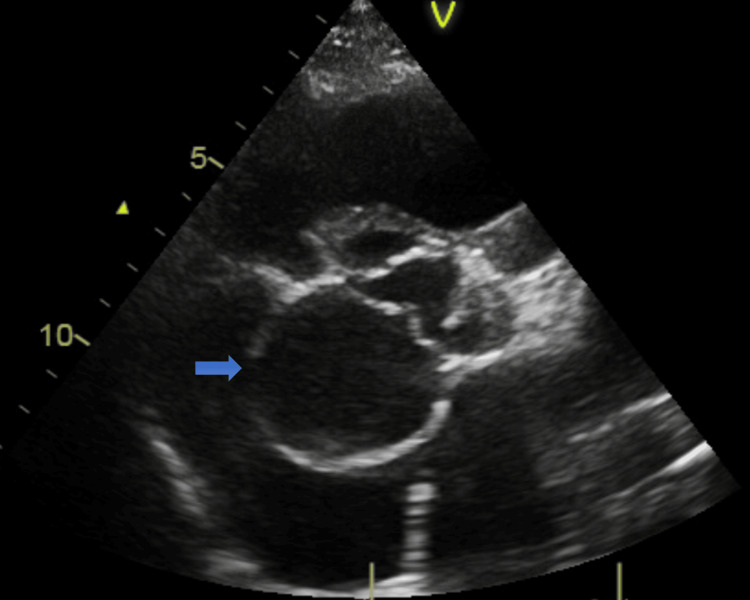
TTE-PSAX view displaying a large SoV aneurysm (blue arrows) TTE, transthoracic echocardiogram; PSAX, parasternal short axis view; SoV, sinus of Valsalva

A transthoracic echocardiogram demonstrated the mass as a possible aortic root aneurysm near the non-coronary cusp with normal left ventricular function. The right ventricle appeared moderately dilated, but its function remained preserved. There was visually eccentric moderate tricuspid regurgitation with a high echocardiographic probability of pulmonary hypertension; worryingly, the estimated pulmonary artery systolic pressure was 73 mmHg (Figure 4).

**Figure 5:**
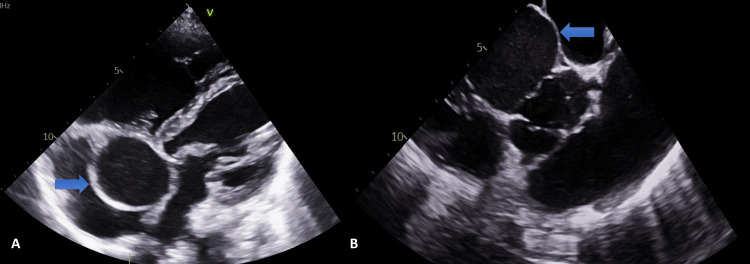
TOE depicting an eccentric aneurysm of the right SoV involving the RCC (A) and protruding into the outflow tract of the right ventricle (B), distorting the Tricuspid valve TOE, Trans-esophageal echocardiogram; SoV, sinus of Valsalva; RCC, right coronary cusp

Transoesophageal echocardiography confirmed an eccentric SOVA affecting the right coronary cusp, with a maximum dimension of 6 cm. We noted a 45-mm mild dilation of the ascending aorta, a small secundum atrial septal defect (ASD) with left-to-right flow, a patent foramen ovale, and a myxomatous mitral valve with P2 prolapse showing mild mitral regurgitation. Although there is left-to-right Doppler flow across the ASD, the patient underwent subsequent investigation for her pulmonary hypertension and cyanosis with the possibility of Eisenmenger syndrome (Figure 5).

Management

A multidisciplinary team spanning emergency, acute medicine, cardiology, radiology, neurology, respiratory, and cardiothoracic surgery, with input from congenital heart disease specialists, managed the patient. Neurological consultation posited that the subependymal nodules were longstanding and potentially related to her epilepsy. The notable amount of white matter disease might stem from chronic microemboli due to her cardiac abnormality. This assumption was because we suspected a cardiac tumor based on CTPA findings alone. Furthermore, the subependymal heterotopia indicated a filamin A gene mutation, which has been associated with cardiac vascular defects [3].

The patient received treatment for a suspected lower respiratory tract infection and fluid overload. With intravenous diuretic therapy and antibiotics, her oxygen saturation improved upon exertion. She was later referred for surgical correction of the SOVA before a complication occurred. As part of the perioperative investigation, she underwent a cardiac MRI and right heart catheterization, which did not confirm Eisenmenger syndrome. During the workup for her pulmonary hypertension, we discovered underlying lung abnormalities in emphysematous changes with mixed obstructive and restrictive patterns on lung function testing. She underwent elective Bentall surgery, which involved mechanical aortic root replacement for the SOVA, repair of the right atrial wall, and closure of the ASD secundum.

Follow-up and outcomes

The patient had a favorable recovery. She could walk on level ground without dyspnea. Examination of her precordium revealed a well-healed sternotomy scar. Postoperative CT scans revealed a stable-appearing aortic root and ascending aorta. She was prescribed warfarin because of her mechanical aortic root. A subsequent appointment with the respiratory team was scheduled to evaluate emphysematous lung changes and pulmonary hypertension further. Unfortunately, genetic testing and counseling were not conducted in her case due to episodes of per vaginal (PV) bleeding, necessitating a total hysterectomy in the subsequent year.

## Discussion

SOVAs primarily affect the right coronary sinus (65% to 85% of cases), followed by the non-coronary (10% to 30%), and, least commonly, the left coronary sinus (<5%) [2]. They can be either congenital or acquired. These aneurysms stem from a weakness in the elastic lamina at the aortic media and annulus fibrosus juncture. SOVAs can be associated with the syndromic fashion of congenital pathologies and connective tissue diseases such as Marfan and Ehlers-Danlos syndrome, bicuspid aortic valve, and other cardiac anomalies like ventricular septal defect and aortic regurgitation. Acquired causes encompass infections (e.g., syphilis, bacterial endocarditis, tuberculosis), atherosclerosis, cystic medial necrosis, chest trauma, vasculitic diseases (e.g., Behcet's disease), and iatrogenic injuries, such as those from aortic valve replacement.

Clinical presentations vary widely, from being asymptomatic to causing sudden death due to rupture. Symptoms can encompass dyspnea, chest pain, hemodynamic compromise, myocardial ischemia from blockage of aortic root blood flow or coronary ostia compression, heart failure, arrhythmias, including atrial fibrillation and third-degree heart block, or more general symptoms such as fatigue [4].

While small SOVAs might be managed with primary or patch closure, the gold standard intervention remains the Bentall procedure, replacing both the aortic root and valve [5]. Given the favorable prognosis post-surgery, early identification and surveillance of SOVA are crucial. Genetically mediated aortic root aneurysms typically exhibit a more aggressive natural history than other ascending aortic aneurysms that do not involve the root. A study from the Yale Aortic Institute demonstrated an increased risk of rupture and dissection for aortic root aneurysms surpassing 5 cm, with mid-ascending aorta risks intensifying beyond 5.25 cm to 5.5 cm [6]. Recent guidelines from the American Heart Association and American College of Cardiology recommend resection timings based on these measurements [7].

FLNA gene is located on Xq28 and encodes the filamin A protein. Mutation in filamin A can result in cell basal body positioning and ciliogenesis, disrupting neuronal migration to the cerebral cortex and cardiovascular defects [2]. Data regarding the morbidity and mortality of cardiovascular complications from loss of function (LOF) FLNA mutations remain limited. It is important to note that not all individuals with filamin A mutation will develop SOVA. A combination of genetic factors and individual susceptibility likely influences SOVA formation. An analysis of 114 patients with LOF FLNA mutations with periventricular nodular heterotopia showed significant cardiac and vascular abnormalities [8]. Although the study's primary focus was thoracic aortic aneurysms, it noted an association with SOVA in many cases. Further research is needed to fully understand the complex relationship between the filamin A mutation and SOVAs. Importantly, it underscored the potential benefit of screening patients with neurological manifestations of LOF FLNA variants for cardiac aneurysms. In our case, early cardiac screening might have accelerated diagnosis, potentially alleviating the patient's symptoms. Given the X-linked inheritance of FLNA deficiency, genetic testing becomes vital for implications in genetic counseling, especially when considering prenatal diagnosis based on identified pathogenic variants [9]. Genetic testing is favorable because it can predict the natural course of malignant aortic disease. More than 70 genes, including FLNA, are implicated in thoracic aortic disease, and mutation of each particular gene can modify their "typical" natural history, which can affect counseling for prophylactic surgery [6], and if the pathogenic variant is identified on genetic testing, cascade testing for at-risk relatives with thoracic aortic imaging and further gene-based management is recommended [7].

## Conclusions

This case underscores the utility of comprehensive imaging in assessing potential filamin A mutations. Collaborative multidisciplinary team efforts prove indispensable in evaluating and managing intricate cases. Actively screening patients showing neurological signs of LOF FLNA variants for cardiac symptoms can expedite diagnosis and intervention. Furthermore, genetic testing is vital for individuals diagnosed with FLNA mutation because of its importance in genetic counseling, particularly in determining the optimal timing for prophylactic aortic root resection before being ruptured, and cascade screening for at-risk relatives. 
